# Caribbean nurse migration—a scoping review

**DOI:** 10.1186/s12960-020-00466-y

**Published:** 2020-03-16

**Authors:** Shamel Rolle Sands, Kenchera Ingraham, Bukola Oladunni Salami

**Affiliations:** 1 Faculty of Nursing, Edmonton Clinic Health Academy, University of Alberta, Edmonton, AB T6G 1C9 Canada; 2 Faculty of Education, University of Alberta, Edmonton, AB T6G 2G5 Canada

**Keywords:** Caribbean, Nurse, Migration, Scoping review

## Abstract

**Background:**

The migration of Caribbean nurses, particularly to developed countries such as Canada, the United States, and the United Kingdom, remains a matter of concern for most countries of the region. With nursing vacancy rates averaging 40%, individual countries and the region collectively are challenged to address this issue through the development and implementation of sustainable, feasible strategies. The aim of this scoping review is to examine the amount, type, sources, distribution, and focus of the conceptual and empirical literature on the migration of Caribbean nurses, and to identify gaps in the literature.

**Methods:**

Identified records were selected and reviewed using Arksey and O’Malley’s scoping framework. A comprehensive search was conducted of eight electronic databases and the Google search engine. Findings were summarized numerically and thematically, with themes emerging through an iterative, inductive process.

**Results:**

Much of the literature included in our study (*N* = 6, 33%) originated in the United States. Publications steadily increased between 2003 and 2016, and half of them (*N* = 9) were journal articles. Many (*N* = 6, 33%) of the records used quantitative methods. The themes identified were as follows: (1) migration patterns and trends; (2) post-migration experiences; (3) past and present, policies, programs, and practices; and (4) consequences of migration to donor countries. More than half (*N* = 11, 56%) of the literature addressed nurse migration policies, programs, or practices, either solely or in part. Several gaps were identified including the need for evaluation of the effectiveness of current nurse migration management strategies and to study policies, trends, and impacts in understudied Caribbean countries.

**Conclusion:**

This review demonstrates the need for future research in key areas such as the impact of nurse migration on health systems and population health. The literature tends to focus on Caribbean countries with higher levels of nurse migration. However, data regarding this phenomenon in other Caribbean countries is needed for a more comprehensive understanding of the plight of the Caribbean region and would answer the call from the International Organization for Migration to study policies, trends, and impacts in understudied Caribbean countries.

## Background

Nursing shortages around the world have led to unprecedented rates of international and interregional nurse migration [1]. Recent findings revealed that 40% of nursing positions in the Caribbean remain vacant, primarily due to nurse migration [2]. As a result, national and global policy debates have been triggered to address increasing rates of nurse migration and its impact on health care [3]. While nurse migration is alleviating some of the demand for nurses in receiving or destination countries, it is creating a greater deficit in regions from which nurses are migrating such as the Caribbean [4].

Researchers have studied the motivating factors, also referred to as push-pull factors, for nurse migration and found that in general nurses migrate for professional growth, financial benefits, and educational advancement [5, 6]. While the pull factors included improved working conditions and better opportunities, push factors include poor remuneration, lack of professional development, and stressful working conditions [6].

Countries such as Canada, the United Kingdom (UK), and the United States of America (USA) are considered the primary countries to which Caribbean nurses migrate [7]. Researchers contend that these countries are contributing to the “brain drain” in donor countries [7, 8]. Subsequently, this brain drain is creating short and long-term challenges such as the depletion of economic investments and a health care deficit in human resources for donor countries [7, 9]. While the detrimental impacts of migration of Caribbean nurses are inarguable, it is maintained that there are benefits, primarily that of remittances [7, 10]. Despite these issues being well researched and documented with reports from organizations such as the Pan American Health Organization (PAHO) [11], this global concern persists with few actionable solutions for regions such as the Caribbean.

A scoping review is helpful for contextualizing the background of this complex phenomenon and to map the literature about this topic. The aim of this scoping review was to examine the amount, type, sources, distribution, and focus of the conceptual and empirical literature on migration of Caribbean nurses and to identify gaps in the literature.

## Methods

The team for this scoping review was comprised of three researchers experienced in scoping review methodology as well as in the substantive content areas of health policy and nurse migration. Our approach was underpinned by a five-stage framework that provided transparency and rigour, increasing reliability through enabling the replication of the search strategy. Scoping reviews are useful for mapping the literature and identifying gaps in the literature [12–14]. The five stages of our methodological framework are discussed below.

Stage 1: During this stage, we identified the research questions. Our research questions were intentionally broad as scoping reviews are intended to summarize large amounts of literature on a topic [14]. The following questions were posed to guide our review:

1. What is the scope of the literature about Caribbean nurse migration including the amount, type, sources, distribution, and focus of the conceptual and empirical literature?
2. What are the gaps in the literature?

Stage 2: This stage involved the identification of academic and grey literature. This was accomplished using a comprehensive search strategy that was developed in consultation with a senior research librarian.

### Inclusion/exclusion criteria

Academic literature was restricted to original (i.e. primary) work. Secondary literature (e.g. systematic or scoping reviews, meta-analysis, or meta-synthesis) were excluded, as were editorials, blogs, opinion pieces, press releases, and symposia proceedings. Grey literature, published and unpublished government and non-government reports and policy briefs, as well as books or book chapters were included. Records whose sample was not exclusive to Caribbean nurses but did contain disaggregated data attributable to Caribbean nurses were included. The full text of the document/article had to be available in English. No date restrictions were applied.

The academic literature was searched through nine electronic databases, including two specific to the Caribbean and the Americas, by combining terms from two themes: (1) Caribbean and (2) nurse migration. Key search terms were used alone and in combination in the title and/or abstract and subject headings as appropriate to yield relevant results.

Grey literature was retrieved via the regular Google search engine using the search statement “nurse migration Caribbean site:.org”. The site:.org limited the retrieval of documents to organizational sites such as Pan American Health Organization (PAHO) and World Health Organization (WHO). A complete literature search strategy can be found in Additional File 1. We identified additional studies through hand searches of the reference lists of relevant articles.

Stage 3: This stage involved selection of the peer-reviewed and grey literature. We used a systematic approach to select the literature. Use of the key search terms yielded 237 records which were imported into RefWorks, a reference management program [15]. Duplicates were identified and discarded. The remaining 174 records were imported into Covidence, a program specifically created for systematic and scoping reviews [16]. Fourteen (14) additional records were identified and deleted. Two researchers, SRS and KI, screened the remaining 160 records in two phases. Phase 1 included the independent screening of the titles and abstracts of the records to determine their relevance to the review’s purpose and research questions. Records deemed relevant by either or both researchers were included in the full-text review. Ninety-nine (99) records were deemed irrelevant to the review and discarded. Phase 2 included the independent screening of the full text of 61 records. Any conflicts regarding the full-text records were resolved through a second review of the discordant records and discussion between SRS and KI until full consensus was obtained. Twenty-four (24) records were finally brought forward for data extraction and charting. Six of the records were excluded during the data extraction process. See the PRISMA flow diagram [17] that was used to report the number of records throughout the process in Additional File 2.

Stage 4: This phase involved extracting and charting the data by SRS and KI. Researcher SRS used Microsoft Word to create the data extraction tool, which included categories such as type of record (original journal articles); research methods (qualitative, quantitative, or mixed method) where applicable; geographic location (country or region); and main findings. Prior to its use, the data extraction tool was reviewed by each member of the research team. The data extraction tool ensured standardization of data extraction and charting across the team [14].

Next, information from the Word document was transferred into the Covidence program, which allowed for additions to the standard template to accurately reflect our research questions. Reliability of the data extraction process defined as consistency with the research questions was ensured through two of the team members (SRS and KI) independently using the tool to extract data and compare results from the first 10 full-text records [14]. Any coding discrepancies were discussed among the team and the tool was refined before proceeding with full data extraction. The data extraction framework can be found in Additional File 3.

Two researchers, SRS and KI, independently extracted the data from a total of 25 full-text records. Accuracy was ensured with the comparison of each reviewer’s independent abstracted data and any discrepancies were discussed to ensure consistency between the reviewers. Seven records were excluded during data extraction. The data from the remaining records were compiled into the single literature review software program, Covidence, then downloaded into Excel spreadsheets in Microsoft Excel software for validation and coding.

Stage 5: SRS and KI collated the extracted data into numerical and qualitative thematic summaries. Frequencies were used to report the numerical data, and narrative synthesis was used to summarize the qualitative data, thus addressing our research questions. Our findings were then analysed in relation to the purpose of our review and research questions. Various gaps in the literature were identified. Possible gaps were not identified a priori but were identified through the literature and our observations of the lack of attention to or absence of specific themes/topics. A quality assessment of the literature was not deemed necessary [12, 18].

## Results

A total of 18 published records met the inclusion criteria and are discussed in our review. Presented below are the amount, type, distribution, and sources of the literature, followed by a numerical and qualitative thematic analysis and a summary of the gaps in the literature.

### Amount, distribution, sources, and type of evidence

The geographical distribution of the 18 records revealed 33% (*n* = 6) were from the United States, 28% (*n* = 5) the United Kingdom, 22% (*n* = 4) Canada, and 6% (*n* = 1) Germany, Trinidad and Tobago, and Switzerland respectively. The records were published between 1985 and 2016, with the greatest number (*n* = 3, 17%) published in 2011. Half were journal articles, four (22%) were thesis dissertations, three (17%) were public health global reports, one was a policy brief (6%), and one was a chapter of an edited book (6%).

One third of the records employed qualitative methods. The most common method (*n* = 8, 38%) was quantitative methods, followed by qualitative (*n* = 7, 33%) and mixed methods (*n* = 4, 22%).

### Numerical thematic analysis

Most (*n* = 14, 78%) of the records did not specify a theoretical framework. Only four (22%) studies identified one or more theoretical frameworks/approaches—social network theory, neoclassical, and neo-Marxist approaches to migration with emphasis on economics, anti-racist feminism, post-colonial and feminist post-structuralism, and a combination of emotional labour, interactive work, structure of regulation, and matrices of domination. Five (28%) records, theses, clearly defined the type of migration. Nurses were the population of interest for each publication. Most (*n* = 9, 56%) of the records offered specification to the training levels of the nurses in their studies. Five (28%) of the records focused on nurses trained as registered nurses (RNs), registered midwives (RMs), and licensed practical nurses (LPNs); two (11%) of the sources studied only RMs; three (17%) studied only RNs; and eight (44%) of the sources did not specify the nurses’ training level. The literature discussed two primary migration patterns, interregional (i.e. Caribbean) migration and international migration. International migration was the dominant (*n* = 14, 78%) pattern identified, with the United States of America (USA) (22%), the United Kingdom (UK) (22%), and Canada (6%) identified as the destination countries. Interregional migration was identified in 11% of the literature; however, the destination countries were not specified.

We sought to identify the sectors of employment pre- and post-migration. Almost 90% of the literature did not report pre-migration employment. One study reported employment in the public sector (6%) and another reported that the nurses were untrained and seeking certification after migration (6%). Just over 50% (*n* = 10) of the literature reported post-migration employment. Approximately 22% (*n* = 4) reported the public sector, and another 22% (*n* = 4) reported a combination of public and private sector employment. Specifically identified Caribbean donor countries included Jamaica (*n* = 2, 11%) and The Bahamas (*n* = 1, 6%). The migration experiences of Jamaican, St. Kitts and Nevis, Trinidadian and Tobagonian, Bajan, Guyanese, and Haitian nurses are discussed (*n* = 11, 61%).

### Qualitative thematic findings

To follow is a summary of the qualitative thematic findings based on four main themes emerging from the literature. The themes include migration patterns and trends; post-migration experiences; past and present policies, programs, and practices; and consequences of migration to donor countries. We close this section by presenting identified gaps in the literature.

#### Migration patterns and trends

The complexity of migration patterns cannot be overstated and may be shaped by political and economic circumstances. Of the 18 records included in our review, 7 (*n* = 39%) provide a snapshot of either past and/or current migratory patterns and trends of Caribbean nurses over the last 60 years.

#### Patterns

Caribbean nurses migrate from rural to urban areas, between Caribbean countries, and most notably internationally [19–21]. While the majority of Caribbean nurses migrate or plan to migrate internationally with the USA, Canada, and the UK as top destination countries, many migrate or plan to migrate to another Caribbean country [21]. Some of these nurses have migrated more than once [22]. At the turn of the century, a staggering 83% of the total stock of registered nurses in the Caribbean had emigrated to industrialized countries [23].

Our review highlights the fact that migration patterns of Caribbean nurses have not changed considerably over time regarding return migration. Many of the nurses who move abroad have no intention of returning to their home country, at least not to work in the nursing profession [22, 24]. Nurses who have expressed a desire to return to their home country plan to return to retire or after retirement from nursing positions abroad [22, 24]. This point may be further compounded when those who do return are not certain that they will remain [20].

#### Trends

Migration trends regarding nurses from the Caribbean are similar in many respects yet demonstrate considerable variance. While the majority of Caribbean nations experience migration of its nursing personnel, it appears that countries such as Jamaica, Haiti, Trinidad and Tobago, and Guyana experience much higher migration rates than other Caribbean countries [20, 21, 25, 26]. These nurses migrate for multifactorial reasons, but primarily for better remuneration (though not always the case), improved work conditions and career advancement, and professional development, also referred to as “push” factors [27]. Nurse migration, while evident, seems to be less problematic in Caribbean countries such as The Bahamas and the Cayman Islands [19, 25]. In fact, next to the three main industrialized destination countries of the USA, UK, and Canada, these islands become destination countries for many nurses from surrounding countries [20, 21, 25]. Interestingly, we found that prior to the turn of the century the main destination country for Caribbean nurses was the UK [21]. However, more recent findings indicate that nurses identified Canada as their main destination country of choice, followed by the USA, and another Caribbean country. The UK was fourth, garnering less than 5% of the responses [20].

Other trends identified through our review included the fact that more experienced nurses, as well as nurses with higher levels of specialization, were targeted for recruitment and eventually migrated; many nurses joined family and friends abroad, who in fact acted as their network for migration and employment opportunities [20]. The latter point may be an emerging trend as it appears that Caribbean nurses are becoming less reliant on recruitment agencies for emigration assistance [22].

#### Post-migration experiences

Half of the records in our review (*n* = 9, 50%) describe the experiences of the nurses once they migrate. The majority of these post-migration records (*n* = 7, 78%) related experiences of how Caribbean nurses were disadvantaged and continue to be disadvantaged through clear patterns of institutionalized racism, marginalization, discrimination, and devaluing of prior credentialing and knowledge [22, 24, 28–32]. Barriers were presented by nursing colleagues, nurse managers, and patients [22, 29, 31, 32]. Some Caribbean nurses were manipulated into accepting enrolled or licensed practical nurse positions instead of registered nurse positions [28] and those who had received education in Canada as opposed to the Caribbean were treated differently.

Caribbean nurses were required to work in subsidiary positions until upgraded. This was not the case for Caribbean colleagues educated in Canada, who often retired in leadership positions, or those educated in Britain who seemed to also enjoy more upward mobility on the job [32]. Caribbean nurses were often relegated to positions in public versus private hospitals, nursing homes, and clinics in New York [24]. These places of employment were seen to have lower occupational status [24]. It has also been suggested that the Caribbean nurses who worked in semi-private hospitals, where salaries were thought to be higher, made similar salaries to their Caribbean counterparts employed in government-operated hospitals and nursing homes [24].

Only one (11%) of the records describing the post-migration experiences of Caribbean nurses offers a comparison between them and those of nurses from the host country. Wheeler et. al [22] found that both groups had similar experiences and noted concerns with the differences in technology in US hospitals and in Caribbean hospitals as well as concerns regarding being given heavier workloads than colleagues. The majority of the Caribbean nurses did not report either exploitation by recruitment agencies or language as an issue upon arrival to the destination country (USA). They did report feeling overwhelmed with having to provide total patient care as opposed to the team nursing care approach and with facing negative responses or reactions to their accented English. The US RNs reported their challenges with understanding the accented English of Caribbean colleagues [22].

In addition to Caribbean nurses’ experiences of struggles, studies in our review relate their experiences of strategizing in response to barriers and discriminatory and racist practices to succeed through individual or group actions. Their resistance and resilience in navigating the dynamics of power relations socially and professionally led to access and integration into their workplaces [28, 29, 31].

Finally, only one (11%) of the studies describing post-migratory experiences of Caribbean nurses discussed the theme of “building ties”. The researchers asserted that at the time of the study, this was a novel finding. It added to the body of knowledge and was relatively new and unexplored in the nursing literature that explored the experiences of overseas nurses in the NHS and indeed throughout the developed world [33].

### Migration policies, programs, or practices

Some assert that the inadequacy of country governments’ policy responses to the root causes of nursing shortages has driven the dynamics of international recruitment. While free trade agreements or blocs may facilitate flows, these only happen when there is a pull-push imbalance, with pull of the destination countries being most important [21, 27]. More than half of the records included in our review (*n* = 11, 56%) discuss various international, regional, and national migration policies, programs, or practices.

#### International

Of the 11 records addressing various migration policies, programs, and practices, only three (27%) discuss how destination countries addressed the immigration of Caribbean nurses. Despite nursing shortages in the 1950s and early 1960s, the Canadian Immigration Department did not want an influx of Caribbean nurses. The immigration department adopted what it called “Women of Exceptional Merit”, which resulted in Caribbean nurses encountering significant barriers in navigating their careers as RNs. These barriers appeared to be related to systemic practices that influenced the regulation of nursing, as well as relationships in work environments [30]. Interestingly, were it not for nursing shortages in Canada, immigration of these nurses would not have been necessary [34].

Technological advancements and an increased budget through the 1965 Medicare and Medicaid legislation in the United States resulted in expansion of the healthcare industry and the changing roles of nurses. These reasons were partly responsible for the need to recruit to fill the growing demand in the United States. Medicaid and Medicare legislature coincided with the Immigration Act in 1965 to increase the emigration prospects of Caribbean nurses. Hence, Nicholson [24] expected and did find the majority (*n =* 125, 89%) of the sample had migrated between the years 1960 and 1980, and more than 75% (*n* = 103) of the nurses were sponsored based on occupation versus relationship.

### Caribbean Forum-European Commission Economic Partnership Agreement

The Caribbean Forum-European Commission Economic Partnership Agreement (CARIFORUM–EC EPA) is meant to achieve three overarching objectives: (1) to alleviate poverty in CARIFORUM; (2) to promote regional integration and economic cooperation; and (3) to foster the eventual integration of the CARIFORUM states into the world economy by improving their trade capacity and creating an investment-conducive environment [35]. CARICOM negotiated greater access for various skilled workers, inclusive of nurses, to European Union (EU) countries on a temporary basis. This agreement came into effect in 2009 and has been heralded as a landmark agreement. However, the effect of such an agreement on stemming nurse migration is questionable. Language constraints largely limit their services to the UK and their services are already in high demand; hence, such programs are not needed to facilitate their access [25].

#### Regional

One (10%) of the records provides insight into numerous initiatives undertaken at the regional level (although discussed in the Jamaican context) to address emigration of nurses and its impacts. The Caribbean Single Market and Economy (CSME) is believed to have the potential to encourage the flow of nurses throughout the region; however, the prospects of better remuneration in traditional destination countries as well as in non-CSME member states such as The Bahamas and the Cayman Islands could thwart expected results [25]. Another policy initiative has been the Managed Migration Program that is supported by the Caribbean Community (CARICOM). This Managed Migration Program includes the economic partnership agreement between CARICOM and the Dominican Republic, which has provided for the temporary movement of skilled persons, including nurses, since 2009 [25]; the Homecoming Program (in 2003), which encouraged Caribbean nurses who practiced abroad to return to their home countries to volunteer and share their skills with local nursing colleagues; and the health and tourism model, which attempted to recruit nurses from destination countries such as Canada, the UK, and the USA to practice in the Caribbean for up to 6 months per year [20].

#### National

Several Caribbean nations have responded to the emigration of nursing personnel by implementing “bonding” of those whose education was subsidized by the government. This policy approach requires nurses to serve the stipulated time of the bond before migrating or repay the cost of their education. Countries have enjoyed varying success, with some recruiting companies opting to pay off the bonds of nurses [25, 36, 37].

Another policy approach adopted by Caribbean countries such as Jamaica and St. Kitts was what some would call “training for export”. This approach would be considered part of the Managed Migration approach, but at a national level, and entails increasing the training of nurses to meet internal and external demands. St. Kitts’ program trains nurses for employment in the USA, and the USA provides reimbursements for training costs [37]. This approach, however, does not appear promising in the Jamaican context with nursing faculty targeted by recruitment agencies, reducing nursing education capacity [25]. What has been heralded as an innovative project is one which allows Jamaican nurses to work in Miami 2 weeks per month while working the remainder of the month in Jamaica [37].

As Caribbean countries grapple with continued nursing shortages, due in part to emigration of their nurses, they form regional and international alliances to recruit nurses to fill vacant positions. The private and public sectors alike engage in regional and international recruitment. Countries like Jamaica and Guyana look to Cuba and India as source countries, although nurses have been recruited from some countries in Africa, such as Nigeria. Intergovernmental agreements between the Jamaican and Cuban governments guide the recruitment of Cuban nurses [25, 34].

### Consequences of migration on donor countries

Approximately one third of the records (*n* = 6) in our review addressed the negative impacts caused by the emigration of Caribbean nurses. In countries such as St. Vincent and the Grenadines (SVG), women enrol in the midwifery program in adequate numbers; however, many do so with the intent to leave the island with a marketable skill, and recruitment agencies target the nurses as soon as they have completed the program [36]. The attrition rate of registered nurses from training programs is sometimes in excess of 20% [23]. The nurses remaining are often adversely affected through even higher patient-nurse ratios and the level and quality of services may be compromised [36]. There are also concerns regarding the impact of the presence of immigrant nurses on the level and quality of services [34]. Many Caribbean countries invest substantially in post-basic training of nurses, the migration of whom translates into a loss in investment resources that they cannot afford [21, 25].

One study describes the loss of nurses from various Caribbean countries to the UK based on nursing council registrations. Haiti, Dominican Republic, and Jamaica were the top three countries identified as losing 6.10%, 2.17%, and 1.03% of country nurse stock as a result of migration to the UK. The Caribbean’s loss totalled over 9% (*n* = 201). Concluding remarks assert that low-income, English-speaking countries involved in high levels of bilateral trade experience greater losses of nurses to the UK [26].

### Gaps in the literature

Several gaps related to the migration of Caribbean nurses, with prospects for future research, have been identified. The literature provides some information regarding migration trends/patterns, post-migration experiences, and strategies to address migration and its impact, as well as consequences of migration to source countries. Several of the Caribbean countries are featured in the literature in varying degrees. However, 61% (*n* = 11) of the literature in our review addresses the migration of Jamaican nurses either as its sole focus or along with other Caribbean countries [20, 23–26, 28–30, 32, 34, 37]. While each Caribbean country should be viewed in the specifics of its context, Pan-Caribbean research comparing and contrasting both patterns/trends and English-speaking Caribbean countries versus non-English-speaking countries could prove useful in the further development of feasible, comprehensive, data-informed policies, programs, or practices to address nurse migration on a regional level. In addition, in the absence of Pan-Caribbean studies, more research is needed to answer the call for “comprehensive, detailed studies on migratory movements, legislation, policy, and other issues” ([38], p. 104) which remain understudied in numerous islands of the Caribbean. Many of the records in our review were cross sectional; hence, no causal relationships could be claimed. More longitudinal studies on Caribbean nurse migration and its impacts would propose causal relationships, and strategies could then be tailored to specifically address relationships.

Although still predominantly female, the male presence is increasing in nursing, albeit more slowly in Caribbean countries. Much of the migration literature pertaining to Caribbean nurses only address the female perspective. A gendered approach would seek to acknowledge and give voice to the experiences of male nurses; then, comparisons between genders could be made. Once again, strategies be they at the regional, national, or organizational level could benefit from data representative of all nurses.

Our review found that while some studies addressed Caribbean nurses specifically, often this group was lumped together with other black or minority nurses, thus requiring disaggregation of data. While the experiences of these groups may be similar in some respects, there are potential differences, even when Caribbean nurses are compared to black nurses from, for example, African countries.

The literature is either silent or very scarce in several areas: (1) Caribbean nurses’ post-migration experiences to another or other Caribbean countries. Future research could offer a comparison of the immigrant nurse’s experiences with host country nurses (and/or internationally educated nurses [IEN]); (2) the perspective of the recruitment agencies regarding their responsibilities and roles in nurse migration and its impacts; (3) the ethics of continued recruitment of nurses from Caribbean islands, which themselves are experiencing severe nurse shortages; (4) the perspective of employers of Caribbean nurses in destination countries of the Caribbean and internationally; (5) remuneration of Caribbean nurses post-migration, specifically in comparison to that of dominant groups/colleagues in destination countries; and (6) the impact of nurse migration on the level and quality of services in countries of the Caribbean.

## Discussion

Our scoping review provides a summary of primary, peer-reviewed, and grey literature related to the migration of Caribbean nurses spanning over 60 years and offers an important contribution to nursing and migration literature through the collating of the relevant research on the topic. A criticism of the literature in our review is the absence of an identified theoretical approach/framework in most of the records. While we acknowledge Vaughan’s caution as cited by Yin [39], p. 35 that the use of theory guides our search, yet simultaneously limits what we find, we also acknowledge that the appropriate use of theory/theories clarifies our understanding of conceptual connections and overall study findings [40]. Use of theory is particularly useful when the area of inquiry is considered complex/multi-faceted [40]. Two or more of the themes were noted in 44% (*n* = 8) of the records in our review, demonstrating the complexity of addressing this phenomenon. The literature is dominated by past and current strategies to address the nursing shortages in Caribbean nations, perhaps in response to calls for country governments to address nurse migration through policies that adequately address the root causes [41–43].

Most of the literature pertaining to nurse migration policies or strategies focuses on the Managed Migration Program of the Caribbean along with interesting country-specific initiatives aimed at minimizing costs while maximizing benefits by managing nurse migration. Salmon et al. [44] also discuss the initiatives we found in our review: The St. Vincent Model: nurses training for export; Jamaican nurses working in Miami: temporary migration—the best of two worlds; the Homecoming Program: brain gain, as part of the “Year of the Caribbean Nurse” (2003); and International nurse recruitment: health and tourism model, and hailed them as country-specific innovations with great potential for developing and maintaining an adequate nursing supply for the Caribbean. Our preliminary assessments indicate that these policies and programs to address migration through short-term strategies have not been very successful or sustainable long term [25, 27, 36]. Murphy et. al. [20] suggest that more is required regarding documentation of the degree to which strategies to manage health care worker migration have been implemented, as well as regarding the analysis of strategies in place for their impacts, if any, or consequences. Countries such as Trinidad and Tobago, Haiti, St. Kitts, Guyana, and especially Jamaica comprise much of the literature, perhaps due to the higher nurse migration levels in the region. However, data from other Caribbean countries could prove useful for cross-sectional analysis and collaboration for possible strategies. IOM [38] suggests further research regarding the policy impacts in the Caribbean, specifically in understudied countries.

The development and implementation of various policies and programs is encouraging; however, what remains less evident is the development and implementation of policies aimed at mitigating “push” factors and the overall improvement of the personal and professional lives of nurses through, for instance, providing better remuneration, improved work conditions, and greater opportunity for professional development and advancement at home. George, Rhodes, and Laptiste [45] found that wage differentials between donor and destination countries are so great that small reductions are unlikely to affect emigration, which suggests that other factors, such as better working conditions and opportunities for professional development and advancement will have to be addressed to influence the decision to migrate. Joint reports from international organizations such as OECD and WHO [46] consistently call for increase in retention efforts, particularly in lower-income countries losing large numbers of their skilled healthcare professionals. Such countries need to attend to some of the “push” factors by increasing their retention efforts by increasing pay rates and improving working conditions [47].

Migration generally, and nurse migration specifically, may be moderated by international and intra-regional policies and agreements in concert with national strategies, making the phenomenon multi-faceted, nonlinear, and reciprocal, as demonstrated in the previous discussion. Caribbean countries act as donor, host, and transit countries to Caribbean nurses, with many of the nurses migrating or intending to migrate internationally [37].

Additionally, previous findings suggest that Caribbean nurses migrate for various personal and professional reasons, with senior specialist nurses targeted by international recruiters [48, 49]. These patterns and trends are similar to those of our review. Future research can include investigating emerging trends, such as the movement of nurses without the aid of recruitment agencies. Additionally, more empirical evidence is needed regarding (1) the effectiveness and (2) the main challenges of policies, programs, and practices currently in use, which is required to inform the development of evidence-based strategies to support and retain Caribbean nurses. However, this could pose numerous challenges since health human resource databases and other migration-tracking mechanisms are either non-existent or functioning at suboptimal levels.

Our review also illuminates post-migration experiences of Caribbean nurses, specifically their encounters with system-wide discriminatory practices, resulting in devaluing, deskilling, and limited upward mobility. In a review of quantitative and qualitative studies from Canada, UK, and the USA, [50] found that internationally educated nurses (IENs) reported frequently experiencing varied forms of discrimination and lack of opportunity in their places of work, with particularly strong evidence in the UK context. Additionally, findings indicated that covert forms of discrimination were more commonly experienced than overt forms [51]. However, the literature does not compare the degree, types, frequency, and responses of male and female nurses to discriminatory practices. This may be the case since male representation in nursing remains comparatively small when compared to female colleagues.

The mass and protracted exodus of Caribbean nurses from the countries in the region has taken a steady toll over time. The major concern articulated through our review and supported by researchers such as George, Rhodes, and Laptiste [45] is the decline of the amount and quality of health care services at all levels including the organizational and national levels. The remaining nursing personnel endure further hardships with increased patient loads and workloads, which may adversely affect the quality of patient care and extend to community and national levels. Additional empirical evidence is imperative to better determine the true extent of migration on society as a foundation on which to develop and implement feasible, sound labour policy planning [25].

While numerous gaps are identified, we suggest more immediate attention to three areas related to nurse migration in the Caribbean: (1) policies, (2) trends, and (3) impacts, particularly, in Caribbean countries previously understudied [38]. Countries such as Antigua and Barbuda, Cuba, Guyana, Saint Vincent and the Grenadines, and The Bahamas are identified as countries lacking comprehensive studies on migration patterns and trends, legislation, and policies [38]. As Dumont and Lafortune [47] note, countries are encouraged to negotiate mutually beneficial agreements in their attempt to better manage health workforce migration; however, the impact of these agreements and related policies requires evaluation. Finally, we support the use of more qualitative research to explore various issues related to nurse migration in the Caribbean, as this method could potentially provide a deeper understanding of migration flows and policies’ impacts and identify policy gaps [38].

### Strengths and limitations

Strengths of our review include that the study was conducted by a team of researchers with expertise in scoping review methodology and international nurse migration and used an exhaustive search strategy with multiple electronic databases including two specific to the Caribbean to identify the peer-reviewed and grey literature. Additionally, we pilot tested and then used standardized screening and extraction forms to ensure consistency in the identification and coding of the data. Two researchers completed the selection of the articles for inclusion as well as data extraction. Despite the rigorous methodological framework utilized, limitations include the exclusion of articles whose full text was not available in English or which could not be located; the possibility of missed literature, particularly grey literature, despite our extensive literature search; and the omission of a quality assessment of the studies [12].

## Conclusion

Our review highlights Caribbean nurse migration. As such, literature in this review focuses on patterns, trends, and past and current strategies from international, regional, and national perspectives of this phenomenon. In addition, through the literature, we synthesize post-migration experiences of Caribbean nurses and the impact of nurse migration on donor countries. This review also demonstrates the need for appropriate use of theory in health workforce research to facilitate a better understanding of concepts and their connections and overall study findings. To address nurse migration adequately, stakeholders must commit to the collection and utilization of empirical evidence regarding the effectiveness of current strategies. Finally, while researchers tend to focus on countries with the greatest levels of nurse emigration, data regarding this phenomenon in other Caribbean countries is needed for a more comprehensive understanding of the plight of the Caribbean region and would answer the call from the IOM [38] to study policies, trends, and impacts in understudied Caribbean countries.

## Data Availability

Any data generated during this study is provided within the manuscript or the additional files.

## Abbreviations

CARICOM: Caribbean Community
CARIFORUM–EC EPA: Caribbean Forum-European Commission Economic Partnership Agreement
CSME: Caribbean Single Market and Economy
EU: European Union
IEN: International educated nurse
IOM: International Organization for Migration
LPN: Licensed practical nurse
OAS: Organization of American States
OECD: Organisation for Economic Co-operation and Development
PAHO: Pan American Health Organization
RN: Registered nurse
RM: Registered midwife
SVG: St. Vincent and the Grenadines
TCN: Trained clinical nurse
UK: United Kingdom
USA: United States of America
WHO: World Health Organization
